# The Role of Real-Time Contrast-Enhanced Ultrasound in Guiding Radiofrequency Ablation of Reninoma: Case Report and Literature Review

**DOI:** 10.3389/fonc.2021.585257

**Published:** 2021-02-23

**Authors:** Rui Zhang, Ming Xu, Xiao-yan Xie

**Affiliations:** Department of Medical Ultrasonics, The First Affiliated Hospital, Institute of Diagnostic and Interventional Ultrasound, Sun Yat-sen University, Guangzhou, China

**Keywords:** reninoma, case report, secondary hypertension, radiofrequency ablation, contrast-enhanced ultrasound

## Abstract

**Background:**

Reninoma is a rare renal endocrine tumor that can cause secondary hypertension, characterized by hypertension, hypokalemia, high renin and aldosterone with normal aldosterone renin ratio (ARR), and occurs more in young female. Mainstream treatment option is surgery, but is less suitable for small or deep lesions, which makes ablation a promising alternative.

**Case presentation:**

Two young female with typical manifestations of reninoma, including hypertension, hypokalemia, high renin, high aldosterone and normal ARR, were treated successfully with real-time contrast-enhanced ultrasound guided radiofrequency ablation, and contrast-enhanced ultrasound was also performed before and after treatment for diagnosis and postoperative assessment. Afterward, their blood pressure and laboratory tests became normal and remained steady during the follow-up of 32 and 6 months, respectively.

**Conclusion:**

Contrast-enhanced ultrasound guided radiofrequency ablations is a promising alternative for reninoma treatment with comparable safety and efficacy with surgery, and has advantages especially in small or deep lesions.

## Introduction

Reninoma, also called juxtaglomerular cell tumor, is a rare cause of secondary hypertension due to the oversecreting renin of juxtaglomerular apparatus. Since the first case reported by Robertson et al. at 1967 (1), nearly 200 cases were described worldwide, and the incidence rate is 0.023% in hypertensive patients at initial diagnosis (2). It often occurs in young adults, especially female, and is characterized by hypertension, hypokalemia, high renin and aldosterone with normal aldosterone renin ratio (ARR). In cases with severe or chronic hypertension, target organ damage was observed, such as proteinuria, cardiomyopathy, and retinopathy (2). Most cases of reninoma were considered benign, though metastases and recurrence cases had been reported (3, 4), and pathological evidence as well as FDG-PET/CT did indicate malignancy sometimes (5, 6). The gold standard of diagnosis is histopathological examination, while clinical evidence combined with laboratory tests and imaging can also lead to diagnosis preoperatively. Treatment modalities include surgery, ablation, and medical therapy. Surgery is the main option, while the effect of medical therapy is very uncertain, and ablation is not widely used but considered a potential alternative (2, 7, 8).

Here we presented two reninoma cases admitted in our hospital in Feb. 2018 and Mar. 2020. Their lesions were tackled successfully with real-time contrast-enhanced ultrasound (CEUS) guided radiofrequency ablation (RFA), and CEUS was also performed before and after ablation for diagnosis and postoperative assessment. The treatment outcomes were followed for 32 and 6 months, respectively. To our knowledge, these two cases were the first to use real-time CEUS guided RFA in reninoma treatment and filled the blank of long-term efficacy of ablation.

## Case Presentation

### Case 1

**Figure 1:**
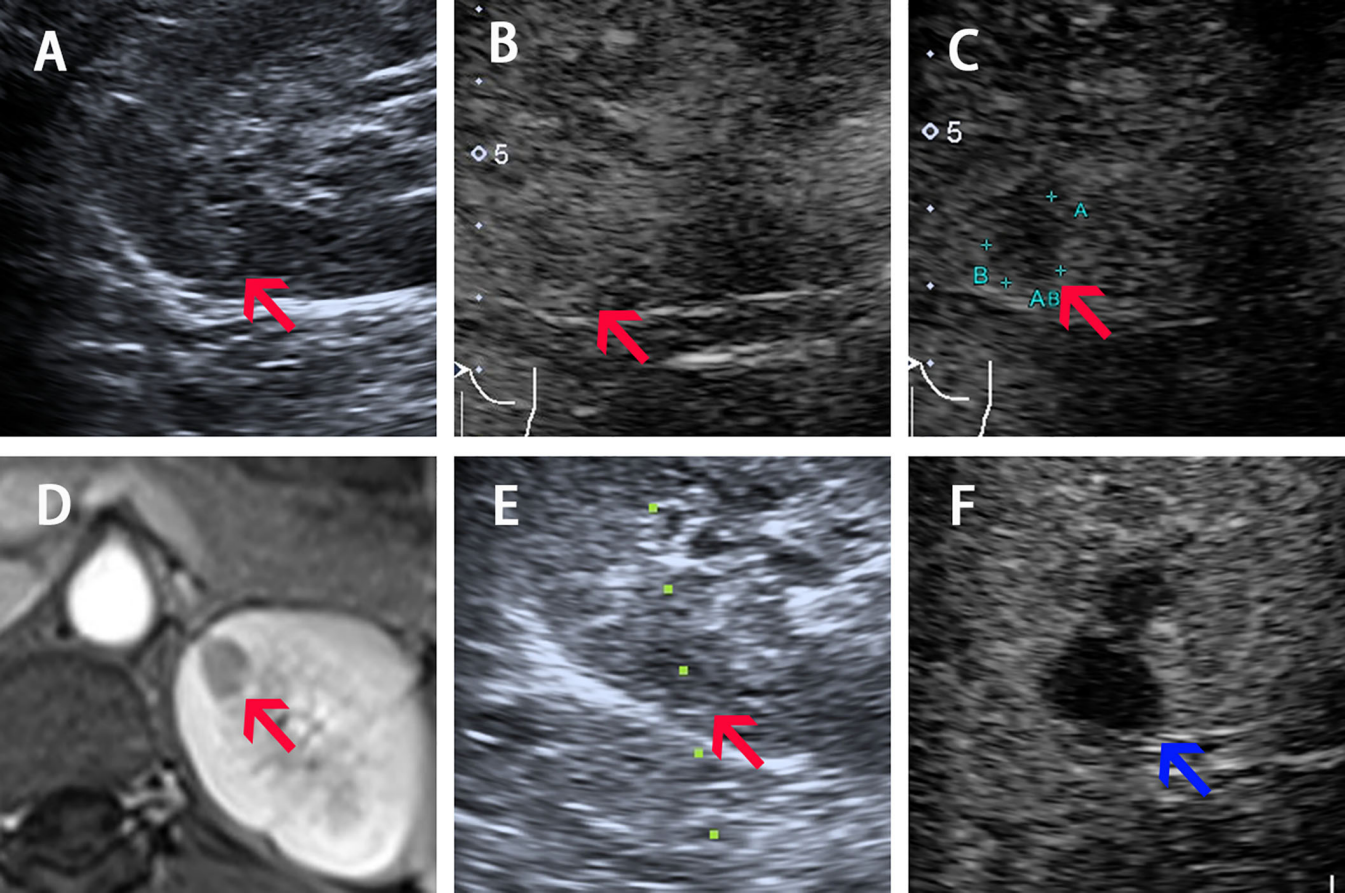
Imaging of Patient 1 before, during, and after ablation procedure. **(A–C)** Ultrasound image acquired in prone position before ablation shows a slight hyperechoic nodule of 10-mm diameter with iso-enhancement in the cortical phase (30 s) and hypo-enhancement in the medulla phase (90 s) (pointed by red arrow). **(D)** Arterial phase of four phases MR before ablation shows a 10-mm diameter nodule (pointed by red arrow) located in the upper pole of left kidney. **(E)** The 17G electrode was implanted into the lesions under real-time CEUS guidance during the ablation (red arrow shows the lesion and the green dotted line shows the puncture path). **(F)** CEUS showed non-enhancement during both cortical and medulla phases postprocedure (pointed by blue arrow).

A 17-year-old female was admitted to our hospital in *Feb. 2018* with paroxysmal headache, nausea and vomiting for over one year, accompanied with marked hypertension (the highest blood pressure was 190/120 mm Hg) and hypokalemia (serum potassium 2.45–3.18 mmol/L). She tried various antihypertensive regimens such as *Spironolactone* 40 mg, *Benazepril* 10 mg combined with *Amlodipine Besylate* 5 mg; *Indapamide* 1.5 mg combined with *Arolol* 10 mg. When taking drugs, her blood pressure was among 120–140/80–100 mm Hg. She discontinued all medication for one month for diagnostic demand. Contrast-enhanced magnetic resonance (CEMR) and CEUS revealed a 10-mm diameter cortex lesion in the upper pole of the left kidney (**Figure 1**). And the key endocrine parameters were summarized in **Table 1**. Renal veins sampling was performed but failed to detect lateralization. Target organ damage evaluation indicated bilateral ocular fundus arteriosclerosis and moderate proteinuria. We gave her *Spironolactone* 60 mg, 10% *Potassium chloride* 45 ml (oral) and *Adalat* 30 mg after admission. The clinical timeline of Patient 1 was organized in **Supplementary Figure 1**.

**Table 1:** Summary of key indicators before and after ablation.

Indicators	Patient 1			Patient 2			Normal range
	Initial admission	After medicine	After RFA	Initial admission	After medicine	After RFA	
Supine PRA (pg/ml/h)	92.7	88.2	–	60.0	46.2	28.1	4.0–24.0
Supine Aldo	186.5	289.19	–	314.82	468.17	272.87	10.00–160.00
(pg/ml)							
Supine ARR	2.01	3.28	–	5.25	10.13	9.71	<30
Upright PRA (pg/ml/h)	163.1	147.1	–	138.3	71.5	47.3	4.0–48.0
Upright Aldo (pg/ml)	209.7	216.26	–	1117.83	1174.6	710.13	40.00–310.00
Upright ARR	1.29	1.47	–	8.08	16.42	15.01	<30
Serum K (mmol/L)	2.55	2.71	3.81	2.95	3.28	3.30	3.50–5.30
BP (mmHg)	144/100	123/80	109/73	184/133	155/108	135/96	<140/90

After RFA, 1–2 days postoperatively; PRA, plasma renin activity; Aldo, aldosterone; ARR, aldosterone renin ratio; serum K, serum potassium; BP, blood pressure; -, undone.

### Case 2

**Figure 2:**
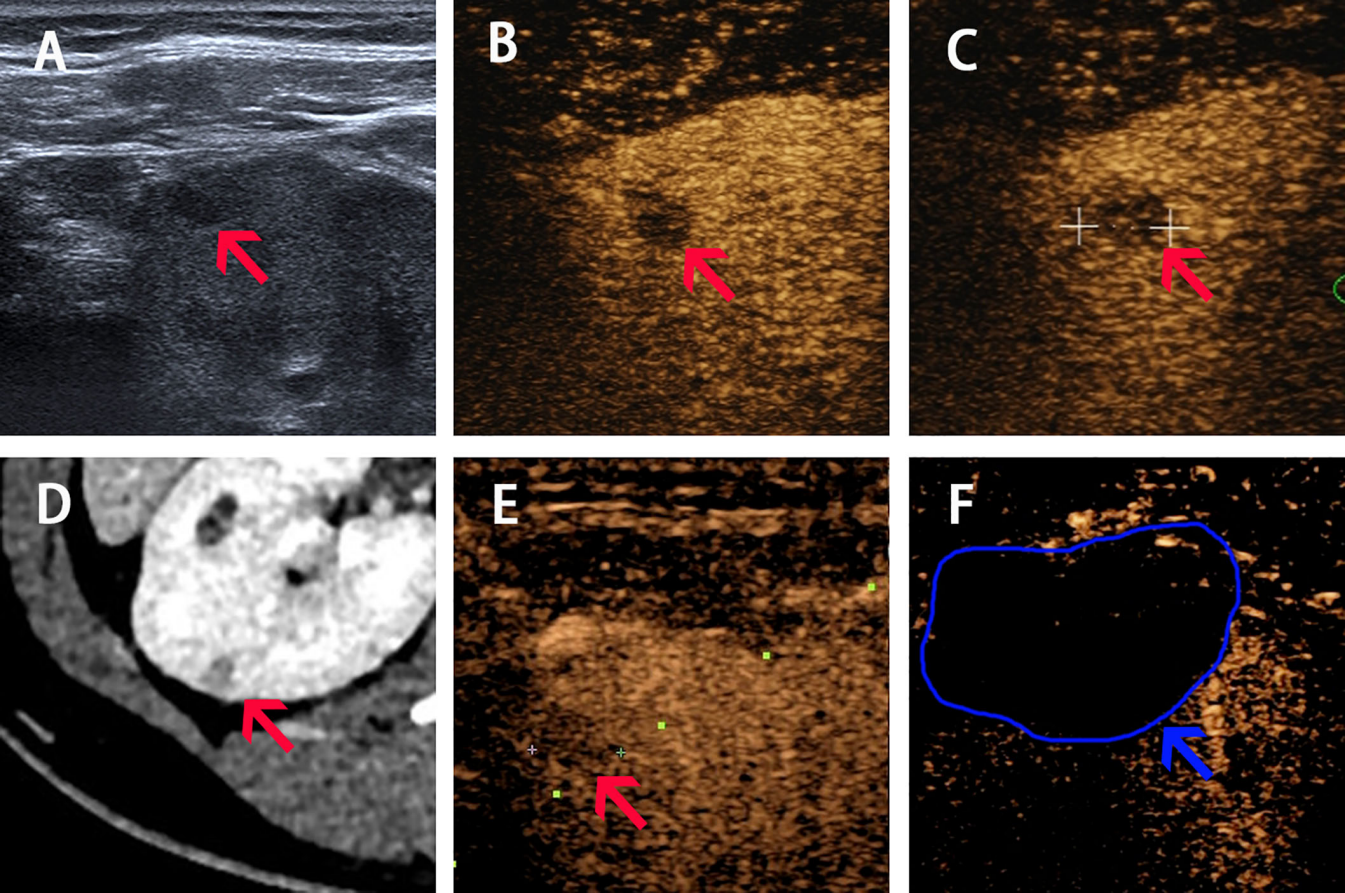
Imaging of Patient 2 before, during and after ablation procedure. **(A–C)** Ultrasound image acquired in prone position before ablation shows a slight hypoechoic nodule of 6-mm diameter with hypo-enhancement in both cortical phase (24 s) and medulla phase (70 s) (pointed by red arrow). **(D)** Arterial phase of CT before procedure shows a 6-mm diameter nodule located in the right kidney (pointed by red arrow). **(E)** The real-time CEUS guided the locating of nodule during ablation (red arrow shows the lesion and the green dotted line shows the puncture path). **(F)** CEUS showed non-enhancement during both cortical and medulla phases postprocedure (pointed by blue arrow and curve).

A 27-year-old female with a 5-year history of poorly controlled hypertension was referred to our hospital in *Mar. 2020*. The highest blood pressure was 179/99 mm Hg. She intermittently used antihypertensive regimens (including *Fosinopril* 10 mg combined with *Metoprolol* 47.5 mg; *Telmisarta* 40 mg combined with *Spironolactone* 40 mg), and her blood pressure was among 120–140/80–90 mm Hg while taking medication, but returned to 170/100 mm Hg after withdrawal, and she stopped taking any medicine for four months for diagnostic need according to the advice of her doctor. Contrast-enhanced computed tomography (CECT) and CEUS revealed a 6-mm diameter cortex lesion in her right kidney (**Figure 2**). Key endocrine parameters were summarized in **Table 1**. Target organ damage evaluation found nothing except slight proteinuria. We gave her *Terazosin Hydrochloride* 4 mg, *Diltiazem Hydrochloride* 90 mg and oral potassium supplement after admission. The clinical timeline of Patient 2 was organized in **Supplementary Figure 2**.

### Multiple Disciplinary Team (MDT) Discussion

MDT discussion was held by endocrinologists, urologists and interventional ultrasound doctors for two patients. Both patients were young female with hypertension, hypokalemia, high renin, high aldosterone and normal ARR. Secondary hypertension caused by endocrine diseases were considered, including pheochromocytoma, Cushing syndrome, congenital adrenal hyperplasia, primary aldosteronism and reninoma. Pheochromocytoma was firstly excluded according to the absence of paroxysmal hypertension or sweating and negative Regitine test in both patients. Cushing syndrome was then ruled out since there were no signs of Cushing appearance and abnormal cortisol level. Congenital adrenal hyperplasia (usually appear as high renin, low aldosterone and decreased ARR) and primary aldosteronism (usually appear as low renin, high aldosterone and increased ARR) were also excluded. Consensus of reninoma diagnosis was finally achieved according to clinical evidence. Both patients refused preoperative biopsy. Surgery and ablation could be the treatment options for both lesions. However, considering that the lesions were rather small and may have difficulty in intraoperative locating, which could prolong the operation time and thus cause more tissue injury and finally damage renal function, percutaneous RFA guided by real-time CEUS was recommended over surgery. And both patients were aware of MDT discussion recommendation and agreed to receive ablation.

### CEUS Examination and Real-Time CEUS Guided RFA Treatment Procedure

The CEUS was performed before RFA. CEUS using a low mechanical index (MI) mode (0.07–0.08) can provide a real-time evaluation of tumor enhancement and location. For case 1, the tumor indicates iso-enhancement and hypo-enhancement during cortical phase and medulla phase. For case 2, the tumor indicates hypo-enhancement in both cortical and medulla phases (**Figures 1** and **2**).

RFA was performed under real-time CEUS guidance using the *Toshiba* ultrasound system. Two physicians who had 10 years of experience performing RFA for renal tumors performed all the procedures. A 20-cm-long, 17-gauge Cool-tip radiofrequency electrode with a 2-cm-long exposed tip (*Covidien Valleylab, Boulder, CO, United States*) was inserted into the targeted tumor. RFA was performed under local anesthesia. The temperature of the ablated tissue was increased to above 60°C and the ablation duration was 12 min for both patients. Immediate CEUS after ablation was done for Patient 2 as shown in **Supplementary Figure 3**. The next day, we performed CEUS for both patients and found the ablation area indicated non-enhancement throughout both cortical and medulla phases, and completely covered the former nodules with satisfactory ablation margin. No intraoperative adverse events occurred and the vital signs remained stable in perioperative period.

### Postoperative Results

The laboratory tests 1 to 2 days after RFA were also summarized in **Table 1**. As for Patient 1, the result was delightful, blood pressure and serum tests became normal 12 h after the procedure without any drugs. Patient 2 had a significant decrease of serum renin and aldosterone, her blood pressure and serum potassium remained abnormal without medication, but indeed better than before. Both patients were discharged 3 days after ablation with no discomfort or complications, Patient 2 got take-home medicine (*Valsarta* 160 mg and *Spironolactone* 20 mg) while Patient 1 did not.

### Follow-Up

For Patient 1, during the 32-month follow-up after RFA, her blood pressure (100–120/60–80 mm Hg), serum potassium and other endocrine tests were totally normal without any medication, urinary protein turned negative, and headache never occurred. Patient 2 stopped *Spironolactone* 2 weeks after RFA, her blood pressure remains in normal range (100–120/75–85 mm Hg) with *Valsarta 160 mg* according to the latest follow-up (6 months after ablation). Her serum potassium, other endocrine tests and renal ultrasound showed completely normal. Long-term effect still demands follow-up for Patient 2.

## Discussion

Reninoma is an uncommon but curable renal endocrine tumor which can cause secondary hypertension. Although it is often considered a benign disease, malignant potentials had shown sometimes (3–6).

To the best of our knowledge, these two cases from our institute were one of the few practices using RFA to treat reninoma, and this is the first report to use real-time CEUS as guidance. Both patients went through RFA successfully, and the postoperative results were delightful. We provided a follow-up of more than 2 years after ablation, which filled the blank of long-term efficacy of RFA. Furthermore, we introduced CEUS into the detecting and follow-up routine, which showed great potential in locating small tumors and indicating the ablation region.

Currently, treatment options for reninoma include surgery, ablation and medical therapy. Medication, such as angiotensin-converting enzyme (ACE) inhibitors, angiotensin II receptor blockers, beta-blockers and *Spironolactone* combined with oral potassium can help control blood pressure and serum potassium (2). However, the use of antihypertensive medications before diagnosis can mask hypokalemia and make reninoma more difficult to recognize (9). And the effect varies from person to person. It is hard to control the blood pressure over years without tackling the oversecreting of renin. Thus, medication is only considered an adjuvant therapy.

Surgery procedures, including nephron sparing partial nephrectomy for superficial or small lesions and radical nephrectomy for deep or large lesions, are the mainstream treatment options. The safety and long-term efficacy have been proved with numerous evidences (2, 7, 8). However, when it comes to small nodules with difficulty in intraoperative locating, surgery may lead to prolonged warm ischemic time as well as operation time, and renal dysfunction consequently. Moreover, using radical nephrectomy to remove deep lesions is in cost of renal dysfunction along with more tissue injury (10, 11).

For these small or deep reninoma, ablation can be an alternative. Ablation for reninoma is not regularly performed currently. Less than five cases of RFA and only one cryoablation have been reported, and none of them provided evidence of long-term efficacy (12–15). However, ablation is actually widely used in other renal tumors, including renal cell carcinoma (RCC), Wilms tumor, adenoma and angioleiomyoma (11, 16). It is considered a type of nephron sparing procedure and recommended mainly in benign tumor or RCC of T1a stage (17). But no randomized controlled trial has been conducted yet to compare ablation and nephron sparing partial nephrectomy in renal tumors, and the long-term follow-up data lacks in ablation cases.

There are several types of ablations, including RFA, cryoablation and microwave ablation (MWA). RFA is used most frequently and the ablation shape can be controlled precisely. It is preferred to surgery for patients with nodules less than 4 cm or intolerable to surgery, and those requiring better postoperative renal function in cases of solitary kidney, bilateral lesions, or chronic renal insufficiency (17). Cryoablation has less application, according to recent literatures, it may be effective for renal tumors larger than 4 cm, but is more time-consuming (18). MWA has never been reported in reninoma treatment, but it is a potential option with many advantages, such as less affected by vascular heat-sink effect and tissue carbonization, more effective in large nodules, and more uniform thermal field distribution compared to RFA (19).

As we mentioned above, RFA has advantages for small or deep lesions. The reasons are as follows: for small lesion, the image-guidance allows quick and precise locating that leads to appropriate needle implanted angle and satisfactory ablation range; for deep lesions, it can preserve better renal function by less tissue injury. What is more, compared to surgery, RFA has less complications, less postoperative pain, shorter hospitalization time with comparable safety and efficacy (10, 11, 16, 20). The limitations of RFA application include nodules in dangerous sites and tumors larger than 4 cm (17). For nodules near the renal hilum, RFA have higher risk of renal pelvis injury, bleeding or infection, and incomplete ablation due to the heat-sink effect (21).

Up to now, all the reninoma cases reported using RFA were under guidance of CT (12–14). This is the first report to use real-time CEUS during procedure. With superiority in real-time, non-radiant and high sensitivity in blood supply detection facilitated by the pure blood pool contrast agents, CEUS shows more potential in intraoperative guidance and postoperative surveillance than CT. What is more, the use of CEUS through the diagnosis, treatment and follow-up routine makes it convenient and easy to compare the change of lesions.

There are also limitations of this report. First, no histopathology evidence was obtained due to patients’ unwillingness; but still, MDT discussion had reached in consensus of reninoma after differential diagnosis. Second, the long-term efficacy of Patients 2 still requires to be further confirmed.

## Conclusion

In summary, we presented two typical cases of reninoma here, which was the first report of utilizing CEUS guided RFA in treating reninoma worldwide. We showed that real-time CEUS guided RFA is safe and effective in reninoma treatment, and it is a promising alternative to surgery especially in small or deep lesions. More data are still warranted to further confirm the long-term efficacy of RFA compared to surgery.

## Data Availability Statement

The raw data supporting the conclusions of this article will be made available by the authors, without undue reservation.

## Ethics Statement

Written informed consent was obtained from the individual(s) and minor(s)’ legal guardian/next of kin for the publication of any potentially identifiable images or data included in this article.

## Author Contributions

Case report design: all authors. Data collecting and patients’ follow-up: RZ and MX. Drafting of the manuscript: RZ and MX. Critical revision of the manuscript: MX and X-YX. All authors contributed to the article and approved the submitted version.

## Funding

Our work is supported by the National Natural Science Foundation of China (81501489)

## Conflict of Interest

The authors declare that the research was conducted in the absence of any commercial or financial relationships that could be construed as a potential conflict of interest.

## Supplementary Material

Supplementary Figure 1 Timeline for Patient 1.

Supplementary Figure 2 Timeline for Patient 2.

Supplementary Figure 3 Immediate CEUS after ablation for Patient 2, showed non-enhancement area during both cortical and medulla phases, which completely covered the former 6mm-diameter nodule with satisfactory ablation margin (pointed by blue arrow and curve).

## Abbreviations

ARR, aldosterone renin ratio; CEUS, contrast-enhanced ultrasound; RFA, radiofrequency ablation; CEMR, contrast-enhanced magnetic resonance; CECT, contrast-enhanced computed tomography; MDT, multiple disciplinary team; MI, mechanical index; ACE, angiotensin-converting enzyme; RCC, renal cell carcinoma; MWA, microwave ablation.
