# Supplementary figures and images for: The Role of Real-Time Contrast-Enhanced Ultrasound in Guiding Radiofrequency Ablation of Reninoma: Case Report and Literature Review
Source: Front Oncol. 2021 Feb 23;11:585257. doi: 10.3389/fonc.2021.585257 (PMC7935754; doi:10.3389/fonc.2021.585257)

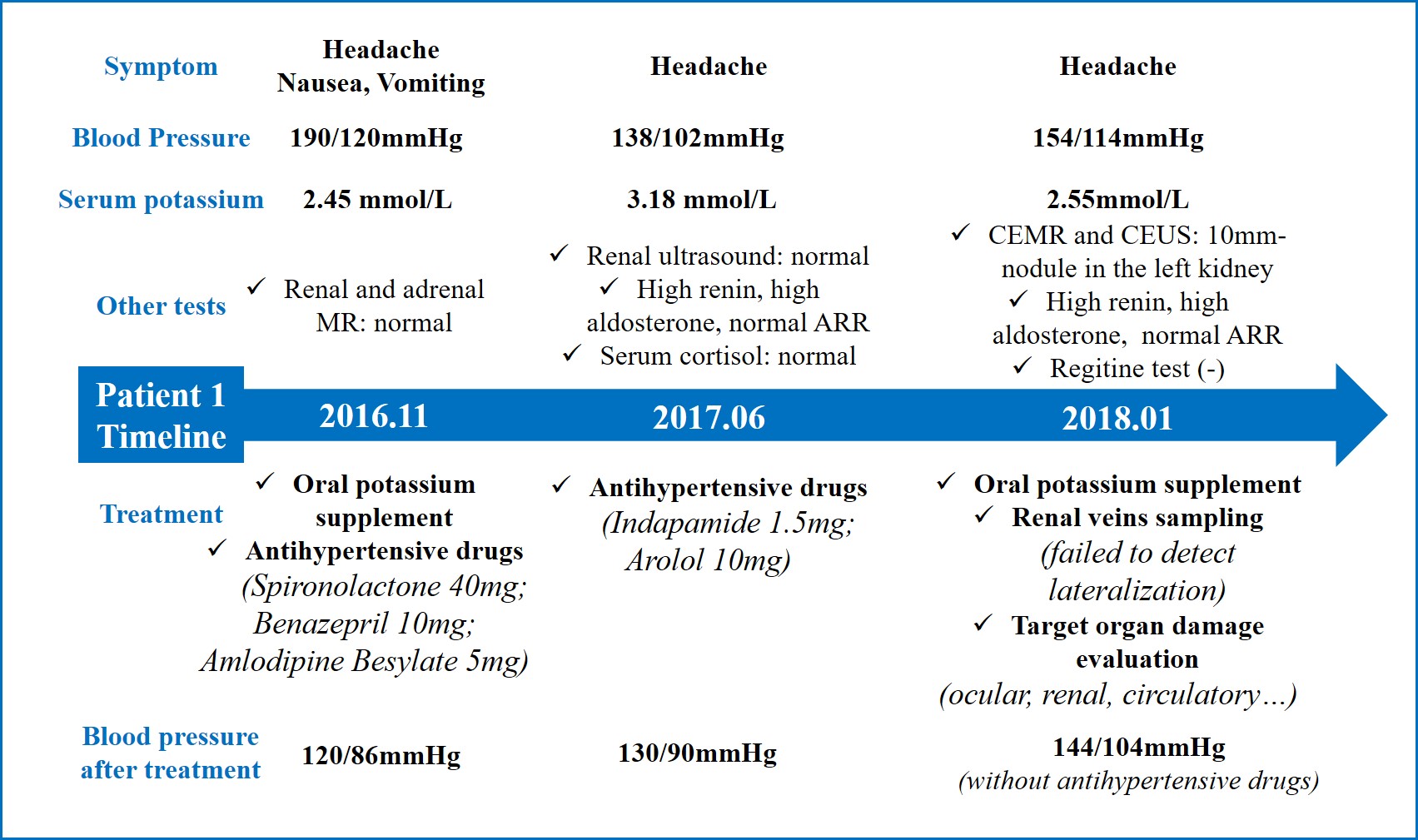

Supplement: Supplementary Figure 1 — Timeline for Patient 1. [file Image_1.jpeg]

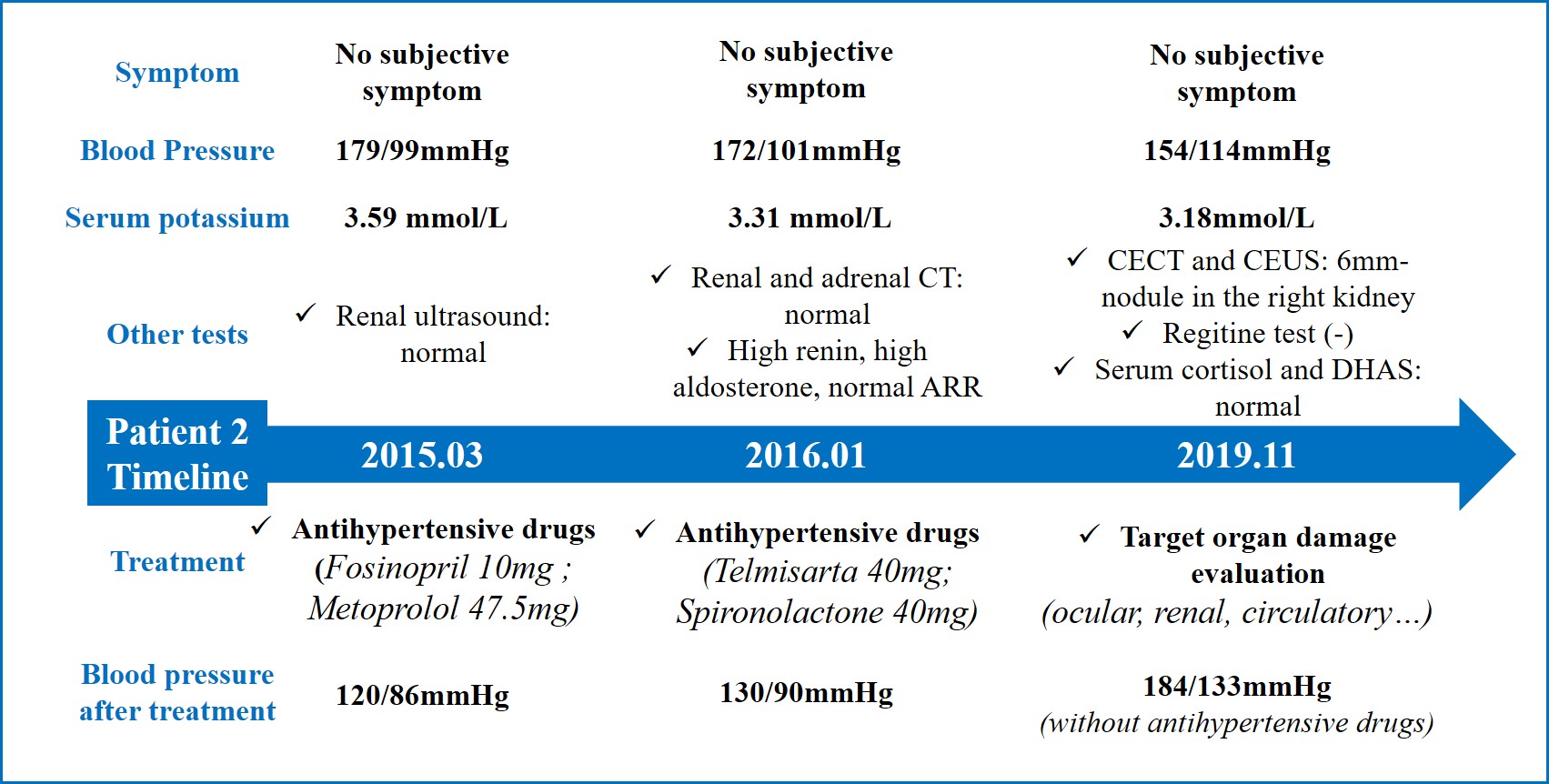

Supplement: Supplementary Figure 2 — Timeline for Patient 2. [file Image_2.jpeg]

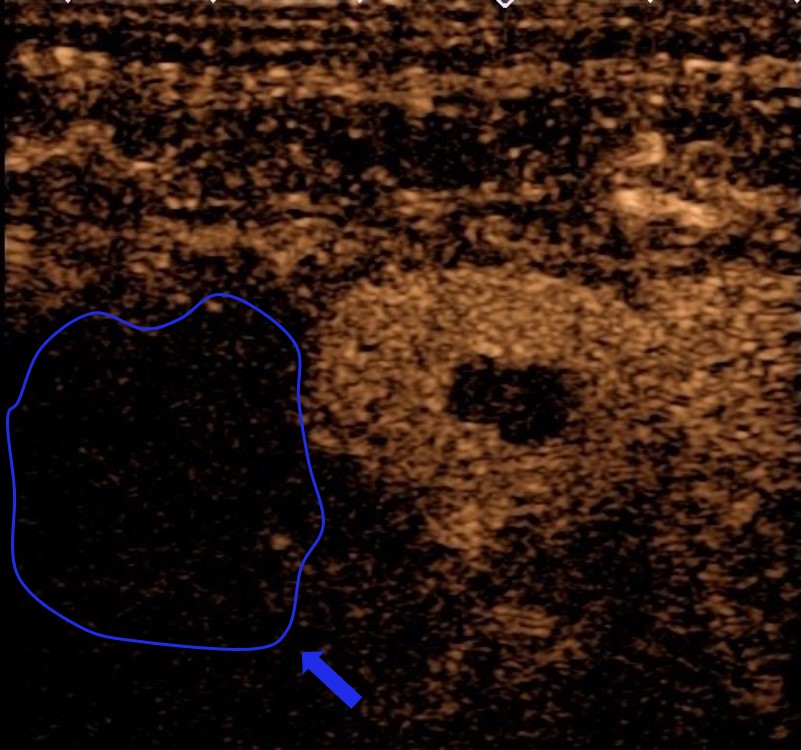

Supplement: Supplementary Figure 3 — Immediate CEUS after ablation for Patient 2, showed non-enhancement area during both cortical and medulla phases, which completely covered the former 6mm-diameter nodule with satisfactory ablation margin (pointed by blue arrow and curve). [file Image_3.jpeg]
